# Health Disparities: The Emerging Trends and Pressing Challenges

**DOI:** 10.3390/ejihpe15010007

**Published:** 2025-01-08

**Authors:** Keren Dopelt

**Affiliations:** Department of Public Health, Ashkelon Academic College, Ashkelon 78211, Israel; dopelt@bgu.ac.il

## 1. Introduction

Health disparities represent one of the most pressing challenges in modern healthcare systems worldwide (Shadmi et al., 2020). The Centers for Disease Control and Prevention (CDC) defines health disparities as “preventable differences in the burden, disease, injury, violence, or in opportunities to achieve optimal health experienced by socially disadvantaged racial, ethnic, and other population groups and communities” (The Centers for Disease Control and Prevention (CDC), 2024). Health disparities have a profound influence on communities and individuals, shaping their overall health, well-being, and social dynamics (Cogburn, 2019). These preventable differences in health outcomes, disease burden, and access to quality care continue to affect socially disadvantaged populations, perpetuating cycles of inequality and undermining the fundamental principle of health equity (Hoagland & Kipping, 2024). As we delve deeper into the 21st century, the urgency to address these disparities has never been more apparent, with emerging trends highlighting both the persistence of long-standing issues and the rise of new challenges in our rapidly evolving global landscape.

The *European Journal of Investigation in Health, Psychology and Education* has dedicated a Special Issue to the exploration of the critical topic of “Health Disparities: The Emerging Trends and Pressing Challenges.” This collection of research aims to shed light on the multifaceted nature of health disparities, their root causes, and potential solutions, bringing together insights from various disciplines to inform policy, practice, and future research directions.

## 2. Understanding Health Disparities

Health disparities are common everywhere, both within countries and between countries (Crimmins et al., 2019; Ravallion, 2018). It has become among the most pressing health challenges, according to different international organizations such as the World Health Organization and non-health bodies such as the UN and the World Bank (Kirigia & Asante, 2021). Ministries of health around the world are preoccupied with measuring health disparities, building interventions, and evaluating them (Mackenbach et al., 2018).

Although this topic is troubling health systems and researchers, health disparities still exist and reflect societies’ structures, historical backgrounds, as well as economic, environmental, and political factors, on top of the approaches of ministries of health (Schillinger, 2020). Solutions have been sought for years, but this wicked problem still persists.

The current volume presents current perspectives on how to measure health inequalities, the fundamental roots for their persistence, and what the approaches for tackling this problem should be. Case studies emerging from different countries and areas, ranging from Brazil to Japan, are crucial as there are no “cut and paste” solutions. Even in a specific country or area, there might be general or specific developments, ranging from very diverse backgrounds, such as in regulation, demography, and climate change, to healthcare delivery and its financing.

The impact of health disparities extends far beyond individual health outcomes. Health disparities shape community resilience, erode trust in healthcare systems, strain resources, and perpetuate cycles of disadvantage across generations (Parenteau et al., 2023). Addressing these disparities requires a nuanced understanding of their underlying determinants and a commitment to systemic change.

## 3. Key Dimensions of Health Disparities

Race and Ethnicity: Racial and ethnic minorities often face disproportionate health challenges due to a complex interplay of socioeconomic factors, cultural barriers, and systemic racism within healthcare systems (Churchwell et al., 2020; Javed et al., 2022). These disparities manifest in higher rates of chronic diseases, reduced access to preventive care, and poorer health outcomes overall (Davidovitch et al., 2013).

Socioeconomic Status: Income, education, and occupation significantly influence health outcomes (Lindberg et al., 2022). Lower socioeconomic status is associated with reduced access to quality healthcare, nutritious food, and safe living environments, all of which contribute to health disparities (Carethers & Doubeni, 2020).

Geographic Location: Rural and urban underserved areas often face unique health challenges, including limited access to healthcare facilities, shortages of healthcare professionals, and inadequate health infrastructure (Dopelt et al., 2021).

Gender and Sexual Orientation: Gender-based health disparities persist in many areas, while LGBTQ+ individuals often face unique health challenges and barriers to care due to discrimination and a lack of culturally competent healthcare services (Lampe et al., 2024).

## 4. Emerging Trends and Pressing Challenges

As we confront long-standing health disparities, new trends and challenges continue to emerge, such as the following:

Technological Disparities: The rapid digitalization of healthcare has the potential to exacerbate existing disparities, as access to and proficiency with digital health technologies may be limited among certain populations (Badr et al., 2024; Richardson et al., 2022; Wilson et al., 2024).

Climate Change and Health Equity: Environmental changes disproportionately affect vulnerable populations, introducing new health risks and exacerbating existing disparities (Bolte et al., 2023; Gutschow et al., 2021).

Global Pandemics: The COVID-19 pandemic has starkly highlighted and, in many cases, worsened existing health disparities, emphasizing the need for equitable public health responses (Dopelt et al., 2024).

Mental Health Disparities: Growing recognition of mental health issues has revealed significant disparities in access to mental health services and outcomes across different populations (Parenteau et al., 2023).

## 5. Addressing Health Disparities: A Multifaceted Approach

Tackling health disparities requires a comprehensive, multi-level approach (Paskett et al., 2016), as follows:

Policy Interventions: Developing and implementing policies that address social determinants of health, improve access to care, and promote health equity across all sectors of society (Gómez et al., 2021).

Healthcare System Reform: Transforming healthcare delivery to be more culturally competent, accessible, and equitable, including diversifying the healthcare workforce.

Community Engagement: Empowering communities to participate in health decision-making and implementing community-based interventions tailored to local needs (Dopelt et al., 2023).

Research and Data Collection: Continuing to invest in research that explores the nuances of health disparities, evaluates interventions, and monitors progress over time.

Education and Awareness: Raising public awareness about health disparities and educating healthcare providers about implicit biases and culturally sensitive care (Dopelt et al., 2014).

## 6. The Role of This Special Issue

This Special Issue of the *European Journal of Investigation in Health, Psychology and Education* serves as a platform for researchers, policymakers, and practitioners to share insights, innovative approaches, and critical analyses related to health disparities. By bringing together diverse perspectives, the issue aims to achieve the following:

1. Raise awareness about the existence, magnitude, and impact of health disparities across various dimensions and populations.
2. Deepen understanding of the complex determinants of health disparities, including social, economic, and environmental factors.
3. Inform policy development and healthcare practice with evidence-based strategies to reduce disparities and promote health equity.
4. Highlight innovative interventions and best practices in addressing health disparities at local, national, and global levels.
5. Identify gaps in current knowledge and priority areas for future research and action.

The collection of articles in this Special Issue spans a wide range of topics, including but not limited to health inequality on local and global scales, health justice and distributive justice, food insecurity, public versus private health insurance systems, health challenges faced by immigrants and minorities, racism and diversity in healthcare, health service utilization among marginalized populations, public health policy, workforce issues in underserved areas, and the broader social determinants of health.

By fostering a multidisciplinary dialog on these critical issues, this Special Issue aims to contribute to the ongoing efforts to reduce health disparities and achieve greater health equity for all populations. As we confront the emerging trends and pressing challenges in health disparities, the insights gathered here will serve as valuable resources for researchers, policymakers, healthcare providers, and community leaders committed to creating a more just and equitable healthcare landscape.

The urgency of addressing health disparities cannot be overstated. As we move forward, it is crucial that we continue to build on the knowledge shared in this Special Issue, translating research into action and working collaboratively across sectors to dismantle the barriers that perpetuate health inequities. Only through sustained effort, innovation, and commitment can we hope to create a world where everyone has the opportunity to achieve optimal health, regardless of their social, economic, or demographic background.
